# Face detection in contextual scenes

**DOI:** 10.1371/journal.pone.0304288

**Published:** 2024-06-12

**Authors:** Jonathan Prunty, Rob Jenkins, Rana Qarooni, Markus Bindemann

**Affiliations:** 1 School of Psychology, University of Kent, Canterbury, United Kingdom; 2 Leverhulme Centre for the Future of Intelligence, University of Cambridge, Cambridge, United Kingdom; 3 Department of Psychology, University of York, York, United Kingdom; Edge Hill University, UNITED KINGDOM

## Abstract

Object and scene perception are intertwined. When objects are expected to appear within a particular scene, they are detected and categorised with greater speed and accuracy. This study examined whether such context effects also moderate the perception of social objects such as faces. Female and male faces were embedded in scenes with a stereotypical female or male context. Semantic congruency of these scene contexts influenced the categorisation of faces (Experiment 1). These effects were bi-directional, such that face sex also affected scene categorisation (Experiment 2), suggesting concurrent automatic processing of both levels. In contrast, the more elementary task of face detection was not affected by semantic scene congruency (Experiment 3), even when scenes were previewed prior to face presentation (Experiment 4). This pattern of results indicates that semantic scene context can affect categorisation of faces. However, the earlier perceptual stage of detection appears to be encapsulated from the cognitive processes that give rise to this contextual interference.

## Introduction

Visual scenes contain statistical regularities such as co-occurring objects and common spatial configurations. Consequently, when objects are expected to appear within a particular scene (e.g., toasters appear in kitchens), they can be detected and categorised with greater speed and accuracy compared to when they are unexpected [1–6]. Object perception is therefore *proactive*, as the visual system is continually generating predictions about the objects it is likely to encounter, based on the surrounding context [7–10]. These processes are also fast. The conceptual gist of a scene context–that is, its global structural and semantic properties–can be extracted from brief presentations (e.g., 20ms) [11–15] and object likelihood predictions may be generated in as little as 80 milliseconds [4].

While scene gist information can influence the rapid processing of objects in general [16], there is also evidence that predictions about *social* objects, such as faces, can be generated from the visual context to influence perception. Impressions of faces, such as judgements about their emotions, traits and social categories can be biased by contextual information from the scene background [17–21]. For example, observers’ judgements of face ethnicity (e.g., White or Asian) are systematically biased toward the cultural context of the scene (e.g., stereotypically American or Chinese settings) [18,19]. This finding demonstrates that some face processing tasks can be influenced by the semantic consistency with the scene background. However, faces were always presented as salient, centrally-presented objects in those experiments. It is not clear whether these effects would extend to the categorisation of faces in peripheral vision [22,23].

One task that requires the perception of faces in the visual periphery is *detection*. Detection is an important process: before a face can be categorized on any dimension, its presence in the visual field must first be determined. It is also a rapid perceptual process [24–26] that is based on general visual properties of faces such as colour and shape [27–33]. For example, when veridical skin colour information is removed from faces or they are rendered in unnatural colour tones [27,31], or the aspect ratio is distorted [29], detection performance suffers.

Such findings suggest that face detection might operate via a template that codes low-level visual information. This might be subserved by a specialized processing channel that facilitates face detection through the activation of hard-wired integration fields that interact with attention [34]. However, recent work indicates that this process is also modulated by differences between *social* categories [27]. In that study, observers were asked to detect faces that varied systematically by ethnicity. Differences in observer’s detection templates, tuned through perceptual experience with faces of different ethnicities, led to ingroup biases in detection performance. If detection templates can be tuned to such stable aspects of our environment, then the question arises as to whether they can also be updated flexibly to reflect expectations about social categories generated from the scene context. The alternative is that performance on face detection tasks is unaffected by scene context. This alternative is plausible. Face detection appears to be fundamentally different to tasks that require face categorisation [22], and the speed and accuracy with which faces can be detected [24–26] may *encapsulate* this process from concurrent contextual information, such as scene gist.

To investigate this question, the current experiments explored whether scene-object semantic congruency can influence the rapid and more elementary task of face detection. We first investigated whether the categorisation of faces (Experiment 1) and scenes (Experiment 2) can be influenced by face-scene semantic congruency. We then explored whether semantic congruency effects extend to the more basic level of face *detection* (Experiments 3 and 4). Specifically, we measured participants’ performance for stimuli in which female and male faces appeared within stereotypically female and male scenes (i.e., clothing shops). Faces appeared without body cues [35], and were distributed systematically across image regions to control for location effects [36]. The semantic scene context was therefore not informative as to *where* the face appeared, but only as to *which category* of face it is–that is, female or male. Although previous work has found that scene context can influence categorisation of centrally-presented faces [18,19] and other objects [4], it was unclear whether contextual influences would extend to faces in the visual periphery.

## Experiment 1

**Fig 1:**
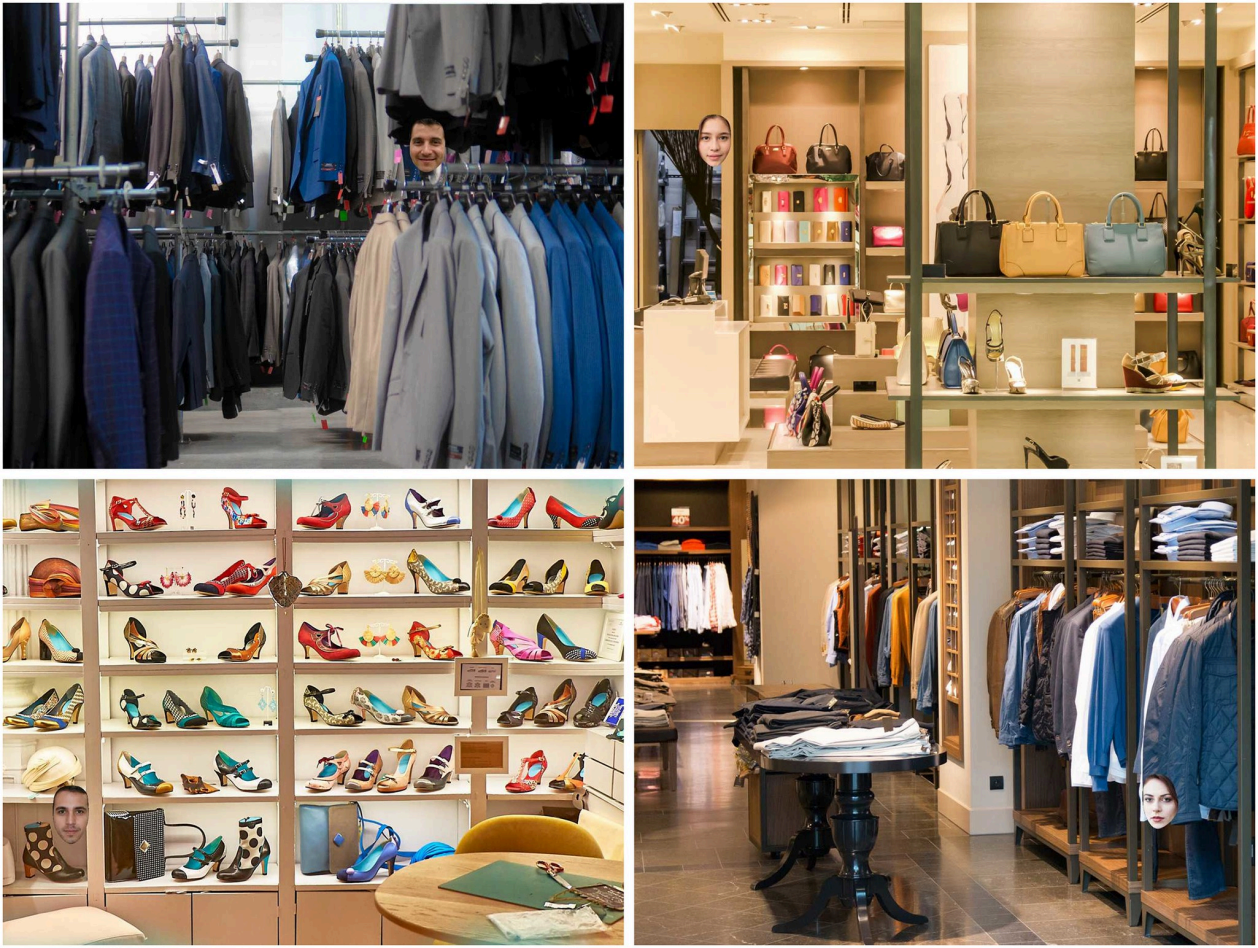
Examples of stimuli used in Experiment 1. Each of the four face-scene congruency combinations are represented: Male face in a male scene (top left); female face in a female scene (top right); male face in a female scene (bottom left); female face in a male scene (bottom right).

In this first experiment, we sought to replicate previous findings that have shown face categorisation to be sensitive to semantic scene context [18,19]. To this end, we systematically manipulated stereotypical female-male congruency of faces and scenes, and measured its effect on face sex categorisation. If semantic congruency matters for face categorisation, then this should be evident in greater response speed and accuracy on congruent trials (i.e., same sex for face and scene) relative to incongruent trials (i.e., different sex for face and scene). By doing this, we planned to extend prior work in two important ways. Firstly, the faces in this experiment were comparatively small, and presented in the visual periphery (see Fig 1)–rather than the large, centrally-presented images used in previous categorisation experiments. This methodological change served to clarify whether scene congruency effects generalise to different viewing conditions. Secondly, Freeman and colleagues [18,19] investigated the effect of scene context on the categorisation of faces by ethnicity, and thus the current experiment sought to extend these findings to the categorisation of faces by sex.

### Methods

#### Participants

A power analysis was conducted with G*Power (Version 3.1) based on a two-tailed repeated-measures t-test with a medium effect size of .5, power of .95, and an alpha threshold of *p* = .05. A medium effect size was chosen for this calculation to reduce the likelihood of Type 2 errors from small samples associated with large effect-size predictions. This suggested a minimum sample size of 54 participants. We rounded this estimate to 60 and adopted this as the sample size for all experiments in this study. For Experiment 1, our sample of 60 participants (30 females; Age *M* = 35.98, *SD* = 8.18) was recruited online using Prolific (www.prolific.co). Aside from demographics, information relating to participant identity was not recorded, and participants were differentiated using an anonymous code provided by Prolific. Recruitment for this study began in December 2021, following ethics approval from the School of Psychology’s Ethics Committee at the University of Kent. All participants were pre-screened to ensure all were in the age range 18–60, were fluent in reading English, and self-reported normal or corrected-to-normal vision. Additionally, participants were screened according to their self-reported ethnicity, ensuring all participants identified as White, given previously-reported evidence that ethnicity can bias detection performance [27]. To monitor data quality, we included eight catch trials to measure participants’ attention, consisting of inverted face-absent scenes that required participants to press ‘Spacebar’. Consequently, five additional participants were excluded: two for failing to reach the 75% threshold on catch trials, and three for incorrect screen calibration.

#### Stimuli

To investigate whether face-scene semantic congruency influences categorisation performance, participants were presented with 160 scene images, all of which contained a face. Half of all faces and scenes were female, and half of all faces and scenes were male. Face-scene combinations included two congruent pairings (female face in a female scene; male face in a male scene), and two incongruent pairings (female face in a male scene; male face in a female scene), with 40 trials each (see Fig 1 for examples).

One hundred and sixty front-facing face images were selected from an existing image set [27], originally sourced from an online face generator (https://thispersondoesnotexist.com). The sex, race and age assignments for this face set were validated by 90 independent observers [27]. For this experiment, we selected faces that were perceived to have the same approximate age (‘young’, i.e., 18–30 years old) and the same race (White), but that differed according to their perceived sex (80 female, 80 male).

Scenes images were comprised of 20 female and 20 male clothing shops sourced from online image repositories (e.g., Unsplash.com, Pexels.com) that provide freely usable images (CC0 license). To validate the female and male assignments of these scenes, we asked 30 independent observers (15 females) to rate each scene as either ‘female’, ‘male’, or ‘no gender’. We found high concordance between observers’ classifications and our category assignments for both female (*M* = 95.61% ‘female’, *SD* = 3.43%) and male scenes (*M* = 95.97% ‘male’, *SD* = 4.27%). To create the 160 experimental trials, each scene was repeated four times: twice with a female face, and twice with a male face.

Faces (143 × 214 pixels) were edited to remove the image background and embedded in scenes (2500 × 1500 pixels). The locations of the faces were determined by a 2 × 2 grid dividing the scene into quarters (1250 × 750 pixels each). For each face-scene category combination, there were an equal number of faces appearing in each of the four regions. Within each region, the precise location of the face was selected at random–aside from defining a 100-pixel border where faces did not appear, preventing placement too near the centre or edge of the scene. Across two counterbalanced versions, each scene was displayed eight times, with one female and one male face appearing in identical locations within each scene region.

#### Procedure

The experiment was conducted online. As stimuli were displayed on participants’ personal computers, we included a screen calibration procedure to ensure that they would be presented at a standard size, by asking participants to adjust an onscreen image to the dimensions of a credit card. Following calibration, scene stimuli were displayed at 21 × 15.75 cm for all participants (visual angle of 19.85° × 14.96°, assuming a 60 cm viewing distance). Faces in scenes were displayed at 1.5 × 2.25 cm (1.43° × 2.2°). Participants were instructed to respond as quickly and as accurately as possible by pressing either ‘F’ or ‘M’, depending on whether they judged the face to be female or male, respectively. Eight attention check trials consisted of inverted scenes and were presented at pseudo-random intervals. When participants encountered an inverted scene, they were instructed to press ‘Spacebar’. Experimental trials were presented in a fully randomised order, with a 1000ms pause between each trial. Participants were also offered the option of a break after 80 trials.

### Results and discussion

The data for this experiment, and all subsequent experiments, can be accessed at https://osf.io/uzcsj/. In all experiments reported here, to gauge whether faces in semantically congruent scenes were categorised more easily than those in semantically incongruent scenes, we recorded the proportion of correctly classified trials as an indication of categorisation accuracy, and median response times (RTs) for correct trials as an indicator of categorisation speed. To summarise the effects of scene context on categorisation performance within a single metric, we also analysed Inverse Efficiency Scores (IES), which are computed by dividing RTs by the proportion of correct responses [37].

**Fig 2:**
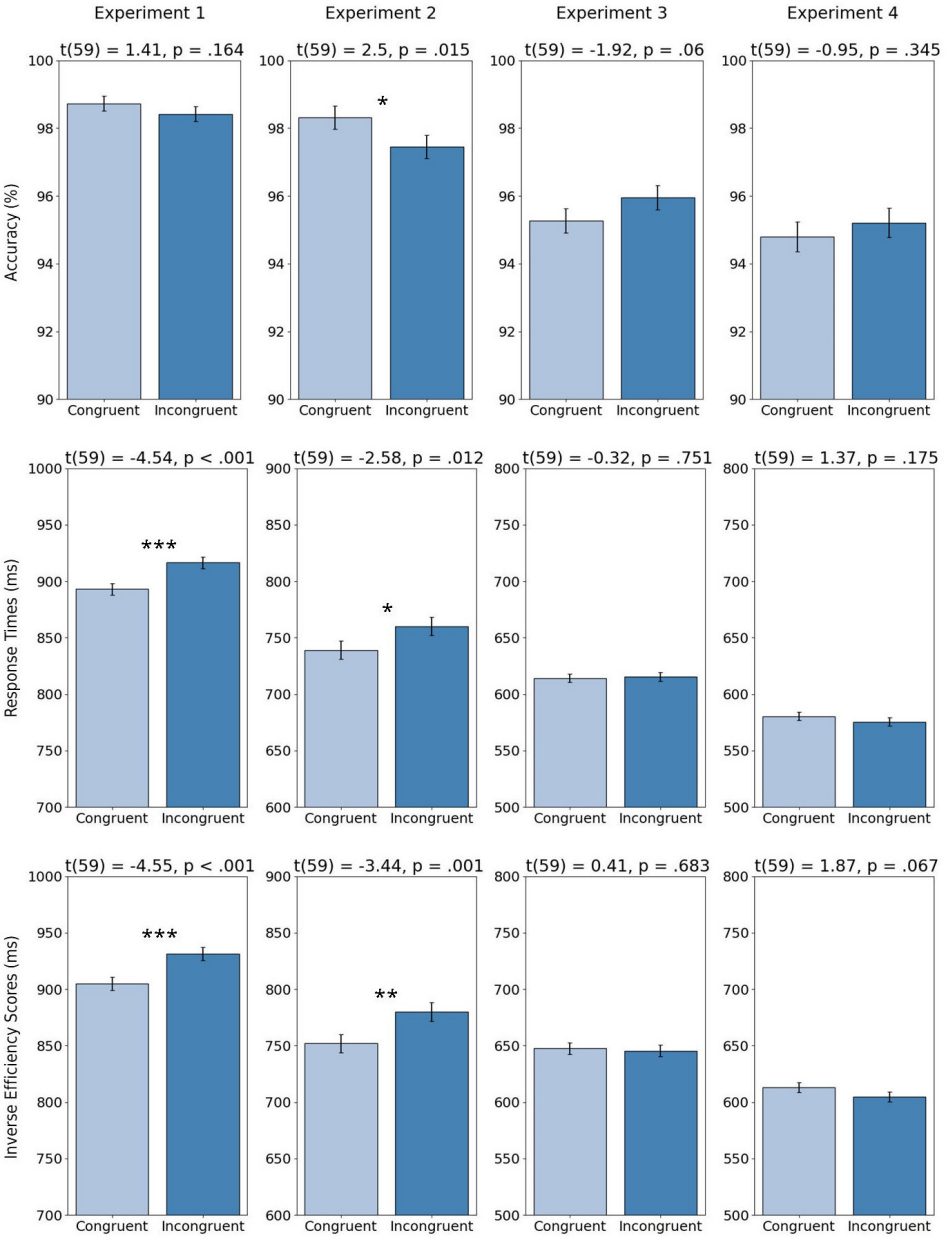
Mean inverse efficiency scores, response times and accuracy for congruent and incongruent face-scene conditions in Experiment 1 (face categorisation), Experiment 2 (scene categorisation), Experiment 3 (face detection), and Experiment 4 (face detection with scene context preview). Above each plot, the results of paired t-tests between conditions are reported. Error bars represent within-subject variability via 95% Cousineau-Morey confidence intervals [38]. *p < .05, ** p < .01, ***p < .001.

We conducted a series of paired t-tests and found clear differences in categorisation performance (see Fig 2). Accuracy scores did not differ between conditions (*M*_*con*_ = 98.73%, *M*_*inc*_ = 98.42%), *t*(59) = 1.41, *p* = .164, *d* = .18, but participants showed faster (RTs: *M*_*con*_ = 893ms, *M*_*inc*_ = 917ms), *t*(59) = 4.54, *p* < .001, *d* = .17, and generally more efficient face categorisation (IES: *M*_*con*_ = 905ms, *M*_*inc*_ = 931ms), *t*(59) = 4.55, *p* < .001, *d* = .19, when faces and scenes were congruent.

These data were also analysed by participant sex (female versus male) with a series of mixed-factor 2 (participant sex) x 2 (congruency) ANOVAs for accuracy, response times and Inverse Efficiency Scores. This did not reveal interactions of participant sex and congruency for accuracy, *F*(1,58) = 1.07, *p* = .305, response times, *F*(1,58) = 1.81, *p* = .184, and Inverse Efficiency Scores, *F*(1,58) = 2.61, *p* = .111. Similar results were obtained for all experiments reported here, indicating that participant sex is not an important moderator of these effects. This exploratory analysis can be accessed in full in the supplementary materials (S1 File).

To investigate the stability of the by-subjects results, we also conducted item-wise t-tests by analysing the accuracy and response times for individual stimulus displays on a cross-subject basis. This showed comparable accuracy for congruent and incongruent conditions, *t*(159) = 1.19, *p* = .235, *d* = .14, but improved categorisation performance on congruent trials for RTs, *t*(159) = 2.69, *p* = .008, *d* = .21, and Inverse Efficiency Scores, *t*(159) = 2.64, *p* = .009, *d* = .22. Together, these results replicate and extend previous work investigating the role of scene context in social categorisation [18,19], by demonstrating that the stereotypical female/male context of the scene background can influence the efficiency of face sex assignments.

## Experiment 2

The previous experiment found that scene background congruency can affect the categorisation of female and male faces embedded in natural scenes. These findings suggest that gist information from the scene context was extracted sufficiently early to either aid or hinder face categorisation, and is congruent with previous reports of rapid gist extraction from natural scenes [12–15,39]. Interestingly, previous work has also shown interference in the other direction: that is, salient foreground objects interfering with scene categorisation [4,40–42]. Consequently, objects and scenes are likely to be processed in parallel, resulting in bi-directional contextual interference. If faces in the visual periphery are detected with sufficient rapidity [34,43,44], the categorisation of scenes might also be better on trials where faces and scenes have semantically congruent category information. In Experiment 2, we tested this assumption by repeating Experiment 1, but instead asked participants to categorise the scene backgrounds as female and male, rather than the sex of the faces embedded within them.

### Methods

#### Participants

Sixty new participants (30 females; Age *M* = 35.03, *SD* = 10.46) recruited on Prolific were included in the final analysis. The same eligibility and inclusion criteria used in Experiment 1 were applied here, and four additional participants were excluded for failing to reach the 75% threshold on catch trials.

#### Stimuli and procedure

Experiment 2 was identical to Experiment 1 except that participants were instructed to categorise the scenes–again by responding ‘F’ for female or ‘M’ for male. Importantly, participants were also instructed to disregard the sex of the face when making their scene judgements.

### Results and discussion

As illustrated in Fig 2, participants’ ability to categorise scenes was influenced by the semantic congruency of an embedded face. When female / male information in faces and scenes was congruent, participants were more accurate (*M*_*con*_ = 98.31%, *M*_*inc*_ = 97.46%), *t*(59) = 2.50, *p* = .015, *d* = .36, faster (RTs: *M*_*con*_ = 739ms, *M*_*inc*_ = 760ms), *t*(59) = 2.58, *p* = .012, *d* = .14, and generally more efficient (IES: *M*_*con*_ = 752ms, *M*_*inc*_ = 780ms), *t*(59) = 3.44, *p* = .001, *d* = .19, at categorising scenes. These effects were also present in item-wise t-tests for accuracy, *t*(159) = 2.78, *p* = .006, *d* = .29, RTs, *t*(159) = 1.92, *p* = .057, *d* = .13, and Inverse Efficiency Scores, *t*(159) = 2.81, *p* = .006, *d* = .20. In combination with Experiment 1, these results suggest that faces rapidly capture attention, leading to bidirectional contextual influences between embedded faces and scene backgrounds during categorisation.

## Experiment 3

The findings from Experiments 1 and 2 converge with those from the object categorisation literature [4,40–42] to suggest that semantic information from faces and scene contexts are processed concurrently, and that their interaction affects social categorisation performance. For embedded faces to influence scene categorisation, faces must have been detected and categorised rapidly by observers–despite being irrelevant to the task–highlighting the automaticity of early face processing. Scene gist extraction was also sufficiently rapid and automatic to influence face perception at the categorisation level (Experiment 1), yet it remains unclear whether the interactivity between face and scene processing extends to earlier perceptual stages–including whether or not a face is present at all. In Experiment 3, we investigated whether scene context effects on face categorisation extend to the level of face *detection*.

### Methods

#### Participants

Sixty new participants from Prolific (30 females; Age *M* = 26.93, *SD* = 8.18) were included in the final analysis, using the same inclusion criteria as the two previous experiments. Three additional participants were excluded for failing to reach the 75% threshold on catch trials.

#### Stimuli and procedure

The primary difference between this and previous experiments was that participants were instructed to judge whether a face was present or absent, rather than make a categorisation decision. Participants were instructed to press ‘P’ (present) if they thought the scene contained a face, and ‘A’ (absent) if they thought it did not contain a face, as quickly and as accurately as possible. To fulfil the demands of the task, an additional 160 face-absent trials were added, forming 320 trials in total. Face-present stimuli were identical to the stimuli used in Experiments 1 and 2, and the same scenes (without faces) were used as face-absent stimuli. We also doubled the number of catch trials to 16, in accordance with the increased total trial number.

### Results and discussion

First, we compared performance on face-present (FP) and face-absent (FA) trials, to determine if participants were adhering to task demands. We found faster responses for face-present trials (*M*_*FP*_ = 614ms, *M*_*FA*_ = 883ms), *t*(59) = 10.22, *p* < .001, *d* = 1.40, which is expected given that visual search is terminated once a face is located. Furthermore, we found greater accuracy on face-absent trials (*M*_*FP*_ = 95.62%, *M*_*FA*_ = 98.29%), *t*(59) = 4.22, *p* < .001, *d* = .54, as participants are more likely to miss a face that is present, than to detect a face where there is none.

Next, we analysed differences between congruency conditions. In contrast to face categorisation, face *detection* performance was not influenced by face-scene semantic congruency (see Fig 2). Paired t-tests between congruency conditions found no differences in detection accuracy (*M*_*con*_ = 95.96%, *M*_*inc*_ = 95.27%), *t*(59) = 1.92, *p* = .06, *d* = .11, RTs (*M*_*con*_ = 614ms, *M*_*inc*_ = 615ms), *t*(59) = 0.32, *p* = .751, *d* = .01, and Inverse Efficiency Scores (*M*_*con*_ = 648ms, *M*_*inc*_ = 645ms), *t*(59) = 0.41, *p* = .683, *d* = .02. Similarly, item-wise t-tests found no congruency effects for accuracy, *t*(159) = 1.43, *p* = .156, *d* = 0.12, RTs, *t*(159) = 0.21, *p* = .833, *d* = 0.01, or Inverse Efficiency Scores, *t*(159) = 1.32, *p* = .188, *d* = 0.09. Unlike categorisation, the present findings therefore suggest that scene context has little influence on detection performance.

Considering the absence of semantic scene context effects on face detection, we explored whether other scene aspects might influence detection, such as *where* faces are likely to be located. For example, as faces are normally attached to the top of a body, they might be located more effectively in upper than lower scene regions. We explored whether such ‘syntactic’ information from scenes might interact with face-scene semantic congruency by conducting a series of 2 (congruency: congruent vs. incongruent) × 2 (location: upper vs. lower scene half) ANOVAs across test items. For accuracy, this showed that faces were detected more reliably in upper than lower scene regions (*M*_*upper*_ = 96.79%, *M*_*lower*_ = 94.44%), *F*(158) = 10.11, *p* = .002, ŋ_p_^2^ = .06. An effect of congruency was not found, *F*(158) = 2.10, *p* = .149, ŋ_p_^2^ = .01, but there was an interaction of location and congruency, *F*(158) = 6.27, *p* = .013, ŋ_p_^2^ = .04. However, contrary to the congruency effects observed in Experiment 1 and 2, post-hoc tests (Bonferroni) shower better detection accuracy for faces in the lower scene regions on *incongruent* than congruent trials (*M*_*con*_ = 93.50%, *M*_*inc*_ = 95.38%), *t*(159) = 2.49, *p* = .030, and no congruency effect was observed for faces in upper scene regions (*M*_*con*_ = 97.04%, *M*_*inc*_ = 96.54%), *t*(159) = 0.87, *p* = .774. In terms of RTs, faces were detected faster in upper than lower scene regions (*M*_*upper*_ = 582ms, *M*_*lower*_ = 656ms), *F*(158) = 42.51, *p* < .001, ŋ_p_^2^ = .21, but there was no significant effect of congruency, *F*(158) = 0.04, *p* = .833, ŋ_p_^2^ < .01, or interaction between these factors, *F*(158) = 42.51, *p* < .001, ŋ_p_^2^ = .21. When accuracy and RTs were combined as Inverse Efficiency Scores, again only a main effect of location was found (*M*_*upper*_ = 604ms, *M*_*lower*_ = 706ms), *F*(158) = 27.78, *p* < .001, ŋ_p_^2^ = .15, indicating more efficient face detection in upper scene regions. There was no significant main effect of congruency, *F*(158) = 1.76, *p* = .186, ŋ_p_^2^ = .01, or interaction between factors, *F*(158) = 2.25, *p* = .135, ŋ_p_^2^ = .01. Together, these results suggest that some factors, such as expectations about location, influence face detection consistently, when semantic cues from the scene context do not.

## Experiment 4

The results of Experiment 3 suggest that face detection might be cognitively impenetrable to the effects of semantic scene context. This would be consistent with prior work, which suggests that face detection and categorisation are qualitatively different tasks [22]. As stimuli were identical to the previous experiments, these results cannot be explained by differences in stimuli, only by the task itself. However, it is possible, given the rapidity of face detection, that these findings reflect differences in processing time between detection and categorisation, rather than the independence of detection from scene context *per se*. If this is the case, detection should show congruency effects when observers are provided with the semantic scene context *prior* to the detection trial. We tested this alternative explanation in Experiment 4.

### Methods

#### Participants

Sixty new participants (30 females; Age *M* = 30.50, *SD* = 10.59) were recruited on *Prolific*, using the same eligibility criteria as the previous experiments. In Experiment 4, eight additional participants were excluded for failing to reach the 75% threshold on catch trials.

#### Stimuli and procedure

In this experiment, we adapted Experiment 3 to provide participants with the scene context prior to the detection trial. In Experiment 2, scenes were classified in 750ms (see Fig 2). Based on these timings, on each trial of Experiment 3 participants were first shown a face-absent scene for 550ms. This was then followed by a 200ms grey mask image, before the same scene image was presented again either with (face-present trials) or without a face (face-absent trials). In this way, the scene context was provided 750ms prior to the detection test, but the 200ms ‘flicker’ was included to disrupt straightforward perception of the change in the scene–that is, the addition of a face on face-present trials [45]. All other aspects of the stimuli and procedure were the same as Experiment 3.

### Results and discussion

As in Experiment 3, we compared participants’ performance on face-present and face-absent trials. Here, we also found faster responses on face-present trials (*M*_*FP*_ = 578ms, *M*_*FA*_ = 796ms), *t*(59) = 8.13, *p* < .001, *d* = 1.13, and more accurate responses on face-absent trials (*M*_*FP*_ = 95.00%, *M*_*FA*_ = 98.44%), *t*(59) = 6.76, *p* < .001, *d* = 1.15, indicating that participants were adhering to task demands.

For our main comparison of interest, we found that participants’ detection performance was not affected by face-scene semantic congruency (see Fig 2), despite providing the scene context prior to detection. Paired t-tests found no differences in detection accuracy (*M*_*con*_ = 94.79%, *M*_*inc*_ = 95.21%), *t*(59) = 0.95, *p* = .345, *d* = .10, detection speed (*M*_*con*_ = 581ms, *M*_*inc*_ = 576ms), *t*(59) = 1.37, *p* = .175, *d* = .06, or Inverse Efficiency Scores (*M*_*con*_ = 613ms, *M*_*inc*_ = 605ms), *t*(59) = 1.87, *p* = .067, *d* = .10. Similarly, item-wise t-tests found no congruency effects for accuracy, *t*(159) = 0.81, *p* = .417, *d* = 0.06, RTs, *t*(159) = 1.19, *p* = .235, *d* = 0.08, or Inverse Efficiency Scores, *t*(159) = 1.27, *p* = .206, *d* = 0.08.

Once again, we investigated whether scene sematic context interacted with participants’ prior expectations about face location by conducting 2 (congruency: congruent and incongruent) × 2 (location: upper and lower) ANOVAs across test items. For accuracy, this showed that faces were detected more often in upper than lower scene regions (*M*_*upper*_ = 96.35%, *M*_*lower*_ = 93.66%), *F*(158) = 9.32, *p* = .003, ŋ_p_^2^ = .06, but there was no effect of congruency, *F*(158) = 0.66, *p* = .418, ŋ_p_^2^ < .01, and no interaction between factors, *F*(158) = 0.80, *p* = .373, ŋ_p_^2^ = .01. Likewise for RTs, detection was faster for faces in the upper scene regions (*M*_*upper*_ = 565ms, *M*_*lower*_ = 604ms), *F*(158) = 19.95, *p* < .001, ŋ_p_^2^ = .11, but the main effect of congruency, *F*(158) = 1.42, *p* = .236, ŋ_p_^2^ = .01, and the interaction, *F*(158) = 0.26, *p* = .609, ŋ_p_^2^ < .01, were not significant. And when these measures were combined as IES, detection was also more efficient for upper-half faces (*M*_*upper*_ = 589ms, *M*_*lower*_ = 657ms), *F*(158) = 15.06, *p* < .001, ŋ_p_^2^ = .08, but no effect of congruency, *F*(158) = 1.61, *p* = .207, ŋ_p_^2^ = .01, and no interaction were present, *F*(158) = 0.23, *p* = .635, ŋ_p_^2^ < .01. Considered in combination with Experiment 3, our results therefore show that face detection remains largely unaffected by semantic cues from the scene context. This is found even though other factors, such as observers’ expectations about the probable locations of faces in scenes, consistently affect detection performance.

### Saliency analysis

After finding that face detection was not influenced by differences in face-scene semantic congruency but by scene location (Experiments 3 and 4), an additional analysis was conducted to explore whether our results may have been influenced also by low-level cues such as image saliency. Within complex scenes, certain visual attributes such as contrast intensity or colour opponency involuntarily attract attention [46,47]. These low-level cues can also interact with high-level semantic information to determine which aspects of a scene are encoded [48]. The visual information within scenes that reliably guide bottom-up attention can be summarised within a saliency map [47]. Here, such maps were generated to quantify the change in visual saliency that occurred once a face was added to a scene region. First, we investigated whether face-saliency differed systematically between congruency conditions within our stimulus set. If saliency were to differ between conditions, then effects of congruency on behavioural performance (Experiments 1–4) may not have been driven solely by high-level semantic information. Second, the role of saliency was investigated more generally, to determine the extent to which visual attention was guided by the low-level visual attributes of scenes.

**Fig 3:**
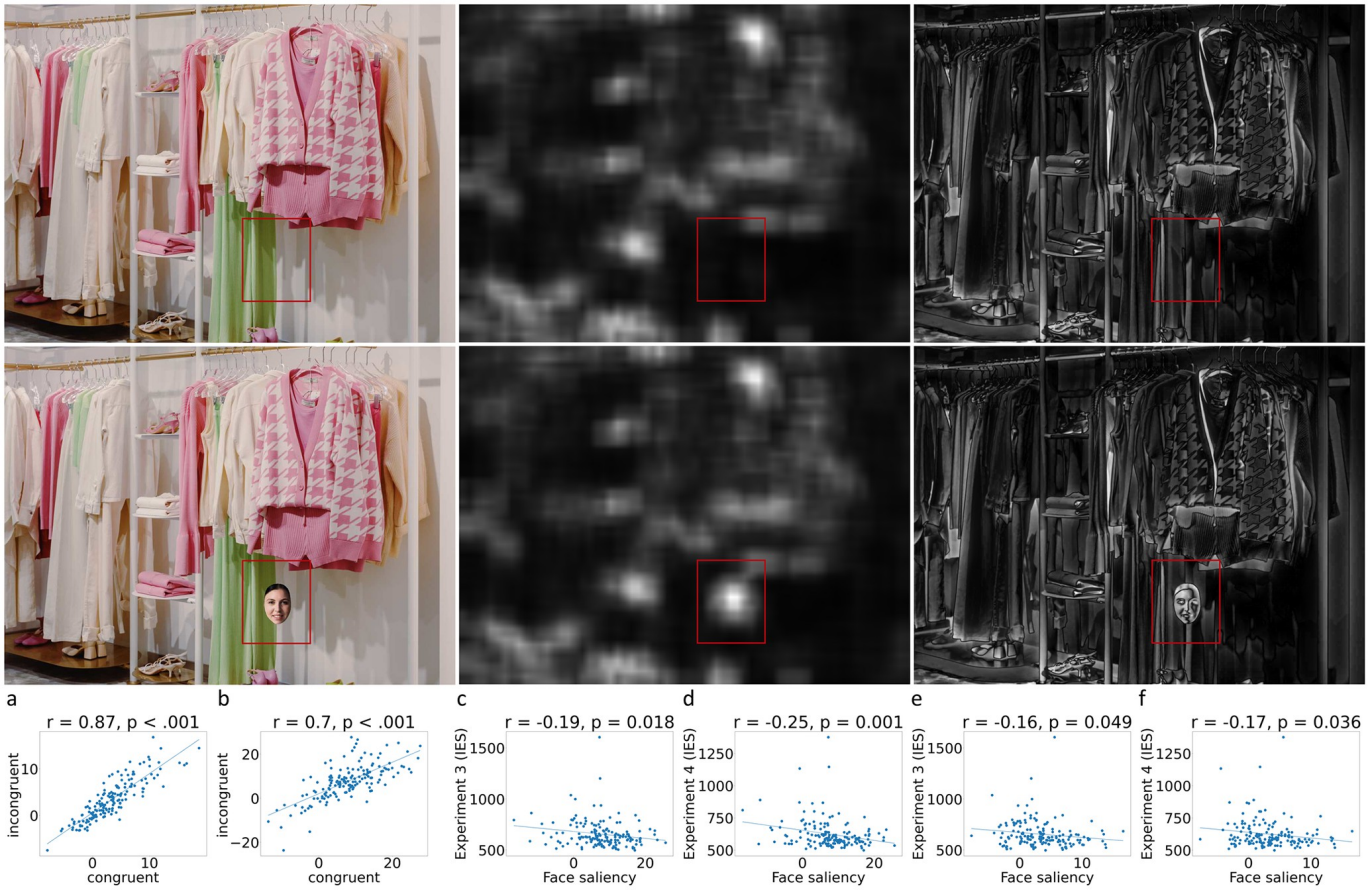
Image saliency for face-present and face-absent scenes (left-hand images) were compared using course-grained (central images) and fine-grained (left-hand images) saliency maps. Face saliency scores were computed by subtracting the sum of pixel values for the face region (red box, lower images) from the corresponding region in face-absent scenes (red box, upper images). Face saliency was highly correlated between congruency conditions for both course-grained (a) and fine-grained (b) methods. Furthermore, face detection performance in Experiments 3 and 4 (Inverse Efficiency Scores [IES]) showed negative correlations with face saliency scores for both course-grained (c and d) and fine-grained (e and f) saliency methods, indicating that salient face regions were detected more efficiently.

Saliency maps for the stimuli of Experiments 1–4 were computed using two separate saliency algorithms, both of which were provided by the ‘OpenCV’ computer vision library and implemented in Python [49]. The first ‘course-grained’ method extracts the spectral residual information from the log-spectrum of an image [50], while the second ‘fine-grained’ method calculates saliency using centre-surround differences inspired by the ganglion cells of the human eye [51]. Example outputs of these two saliency algorithms can be viewed in the central and right-hand panels of Fig 3, respectively.

After generating course- and fine-grained saliency maps for each of the stimuli used in Experiments 1–4, we then focused our analysis on a 180 × 200 pixel region of the saliency map surrounding the location of the face, and its equivalent in face-absent scenes (see Fig 3). We then calculated the sum of the pixel intensities for these regions, and computed the difference between corresponding face-absent and face-present scenes. In this manner, we produced a dataset (*N* = 160) corresponding to the change in regional saliency after the addition of a face for both congruent and incongruent conditions. We first compared differences in saliency between these conditions, before comparing saliency values to the behavioural data collected in detection Experiments 3 and 4.

The change in visual saliency that resulted from adding a face to an image did not differ between semantic congruency conditions for course-grained, *t*(159) = 0.49, *p* = .627, and fine-grained approaches, *t*(159) = 0.08, *p* = .940, and was also strongly correlated in both, *r*(158) = 0.70, *p* < .001 and *r*(158) = 0.87, *p* < .001 (see Fig 3a and 3b). This demonstrates that faces, regardless of their sex, showed highly similar saliency scores when placed in identical locations of the same scene. It also indicates that the detection and categorisation results presented in the preceding experiments are unlikely to be caused by systematic differences in visual saliency between conditions.

After establishing that saliency scores were highly similar for congruent and incongruent trials, we collapsed saliency scores across conditions by computing the mean. We then compared the combined scores to the behavioural data in Experiments 3 and 4, to determine whether face detection performance was influenced by bottom-up image saliency [46]. For the course-grained spectral residual method, face saliency scores were positively correlated with detection accuracy for Experiment 3, *r*(158) = 0.14, *p* = .033, and Experiment 4, *r*(158) = 0.23, *p* = .004. We also found negative correlations between face saliency and participants’ response times in Experiment 3, *r*(158) = -0.21, *p* = .007, and Experiment 4, *r*(158) = -0.28, *p* < .001. In addition, the combination of these measures in the form of Inverse Efficiency Scores (IES) were also negatively correlated with face saliency in Experiment 3, *r*(158) = -0.19, *p* = .018, and Experiment 4, *r*(158) = -0.25, *p* = .001 (Fig 3c and 3d).

For the fine-grained centre-surround method, face saliency also a showed positive correlation with detection accuracy for Experiment 4, *r*(158) = 0.17, *p* = .036, but the effect was marginal for Experiment 3, *r*(158) = 0.14, *p* = .069. However, negative correlations between face saliency and RTs were found for both experiments, *r*(158) = -0.19, *p* = .018 and *r*(158) = -0.17, *p* = .034. Finally, the corresponding IES were also negatively correlated with face saliency for both Experiment 3, *r*(158) = -0.16, *p* = .049, and Experiment 4, *r*(158) = -0.17, *p* = .036 (Fig 3e and 3f).

Taken together, the saliency analysis indicates that the low-level visual saliency of a face within a scene affects detection performance, as more salient faces tend to be detected with greater speed, accuracy, and efficiency relative to less salient faces. Nevertheless, we found no evidence for visual saliency differences between congruency conditions, suggesting that any behavioural differences between these conditions were not driven by saliency cues.

## General discussion

The findings of Experiment 1 and 2 demonstrate that face and scene perception are intertwined, whereby the semantic congruency of scene context influenced the sex categorisation of faces, and vice versa. This demonstrates that social category information about faces and scenes is processed in parallel and integrated in real time, even when faces are presented in the visual periphery or are irrelevant to the task, and extends the growing body of work reporting bidirectional effects of scene-object congruency [4,23,40–42,52] to the rapid processing of these *social* objects. This finding is of general relevance to face perception research as these stimuli are often studied in isolation, separated from their natural scene context [17–21,53]. It also converges with theoretical approaches predicting that semantic expectations generated from visual context can influence early perceptual processing [7–10,54,55].

The question arises as to whether the interference effects from female and male scene contexts in Experiment 1 and 2 extend beyond binary classifications of biological and physiological sex differences between faces. The social construct of gender, for example, refers to greater diversity between people than sex. In this study, female and male information in scenes was also socially constructed and is therefore more representative of gender than sex. The observation that the gender of scene contexts influenced the sex categorisation of faces, and vice versa, therefore suggests that these effects are transferable–at least in some cases–between biological and social information.

However, Experiment 3 and 4 also show that face *detection* is not subject to the same contextual influences as face categorisation, as the search for these targets was not affected by the congruency of the semantic scene gist with face sex. Face detection is a highly rapid perceptual process, that is seemingly robust to natural variability in facial appearance [26,44,56]. Faces also appear to ‘pop-out’ in visual search grids, which suggests that they are detected using a parallel, pre-attentive search strategy, even outside of their natural context [25,57,58]. Thus, face detection might be encapsulated from cognitive processes that can interfere with higher-level face decisions. Alternatively, while the cognitive mechanism underlying detection is sensitive to differences in ethnicity, based on a participants’ exposure to faces from different groups [27], it may be ‘sex agnostic’ given the approximately equal representation of female and male faces in our visual diet.

Although there was no effect of *semantic* gist on detection performance in Experiment 3 and 4, *some* information about the visual context was extracted during detection, as faces were located faster and more accurately in the upper than the lower regions of scenes. This finding might reflect expectations about face locations, whereby faces are located more effectively in scene regions where they might typically appear, attached to the top of a body. This converges with other studies in which *syntactic* gist–that is, the relative locations of scene elements–can aid the selection of likely target locations [59–63], and in which detection is affected by the location at which faces are presented in the visual field [36]. The finding that location affected detection in the current experiments when semantic congruency did not, strengthens the conclusion that detection is encapsulated against interference from scene gist.

While face detection might be encapsulated from cognitive processes such as the processing of semantic gist, there are also alternative explanations for the absence of semantic congruency effects on face detection performance. For instance, there are cortical constraints on perception in the visual periphery, such as reduced acuity and crowding [64,65]. These constraints may limit the influence of face sex information presented in the visual periphery on detection [34,66], and previous work has already shown that scene context can influence categorisation of centrally-presented faces [18,19]. Therefore, semantic congruency effects from stereotypically female and male scenes might emerge when faces are presented foveally. However, this presentation would also eliminate important characteristics of the detection process, such as the search for face candidate regions in the periphery [22,53]. It also remains possible that other contexts might facilitate face detection in contrast to the null effects that were observed with stereotypically female and male scenes in Experiment 3 and 4 here, for example, such as busy inner city versus remote natural environments [27]. As the current experiments investigated semantic face-scene associations based on sex categories only, future work could explore the relative contributions of a wider range of semantic scene gists on face detection.

## Supporting information

S1 File Additional analyses by participant sex. (DOCX)
