# Supplementary material for: Face detection in contextual scenes
Source: PLoS One. 2024 Jun 12;19(6):e0304288. doi: 10.1371/journal.pone.0304288 (PMC11168631; doi:10.1371/journal.pone.0304288)
Supplement: S1 File — (DOCX) [file pone.0304288.s001.docx]

**Additional Analyses by Participant Sex**

In response to a reviewer query, we explored potential effects of participant sex with a series of 2 (participant sex: male, female) x 2 (congruency: congruent, incongruent) mixed-factor ANOVAs for accuracy, response times (RTs) and inverse efficiency scores (IES) for all experiments. We note that we had no *a priori* predictions about participant sex. We summarize these analyses here.

**Experiment 1**

Mean and standard deviation for accuracy (Acc), response times (RTs) and inverse efficiency scores (IES) for the congruent (C) and incongruent (I) condition in male and female participants of Experiment 1.

|  | | | | | | | | | | | | | | | |
| --- | --- | --- | --- | --- | --- | --- | --- | --- | --- | --- | --- | --- | --- | --- | --- |
|  |  |  |  |  |  |  |  |  |  |  |  |  |  |  |  |
|  | | **Sex** | | **Acc C** | | **Acc I** | | **RTs C** | | **RTs I** | | **IES C** | | **IES I** | |
| Mean |  | Female |  | 98.875 |  | 98.333 |  | 866.27 |  | 896.77 |  | 876.45 |  | 911.97 |  |
|  |  | Male |  | 98.583 |  | 98.500 |  | 919.73 |  | 936.37 |  | 933.69 |  | 950.76 |  |
| Standard deviation |  | Female |  | 1.7167 |  | 1.8666 |  | 106.01 |  | 121.39 |  | 108.59 |  | 121.79 |  |
|  |  | Male |  | 1.7287 |  | 1.5536 |  | 165.61 |  | 148.01 |  | 170.55 |  | 149.85 |  |
|  | | | | | | | | | | | | | | | |

Accuracy: ANOVA did not show a main effect of Congruency, *F*(1,58) = 1.99, *p* = .164, or of Participant Sex, *F*(1,58) = 0.03, *p* = .872, and no interaction between these factors, *F*(1,58) = 1.07, *p* = .305.

RTs: ANOVA showed a main effect of Congruency, *F*(1,58) = 20.94, *p* < .001, due to faster responses on congruent than incongruent trials, but not of Participant Sex, *F*(1,58) = 1.76, *p* = .190, and no interaction between these factors, *F*(1,58) = 1.81, *p* = .184.

IES: ANOVA showed a main effect of Congruency, *F*(1,58) = 21.23, *p* < .0001, due to lower IES scores on congruent than incongruent trials, but no main effect of Participant Sex, *F*(1,58) = 1.81, *p* = .183, and no interaction between these factors, *F*(1,58) = 2.61, *p* = .111.

**Experiment 2**

Mean and standard deviation for accuracy (Acc), response times (RTs) and inverse efficiency scores (IES) for the congruent (C) and incongruent (I) condition in male and female participants of Experiment 2.

|  | | | | | | | | | | | | | | | |
| --- | --- | --- | --- | --- | --- | --- | --- | --- | --- | --- | --- | --- | --- | --- | --- |
|  |  |  |  |  |  |  |  |  |  |  |  |  |  |  |  |
|  | | **Sex** | | **Acc C** | | **Acc I** | | **RTs C** | | **RTs I** | | **IES C** | | **IES I** | |
| Mean |  | Female |  | 98.625 |  | 97.667 |  | 692.40 |  | 717.97 |  | 701.72 |  | 735.66 |  |
|  |  | Male |  | 98.000 |  | 97.250 |  | 785.87 |  | 802.36 |  | 802.21 |  | 824.66 |  |
| Standard deviation |  | Female |  | 1.8084 |  | 2.7016 |  | 91.530 |  | 106.64 |  | 89.268 |  | 112.49 |  |
|  |  | Male |  | 2.1676 |  | 2.7738 |  | 162.38 |  | 189.51 |  | 166.45 |  | 190.07 |  |
|  | | | | | | | | | | | | | | | |

Accuracy: ANOVA showed a main effect of Congruency, *F*(1,58) = 6.14, *p* = .016, due to higher accuracy on congruent than incongruent trials. A main effect of Participant Sex, *F*(1,58) = 1.03, *p* = .315, and an interaction between these factors were not found, *F*(1,58) = 0.09, *p* = .764.

RTs: ANOVA showed a main effect of Congruency, *F*(1,58) = 6.59, *p* = .013, due to faster responses on congruent than incongruent trials, and of Participant Sex, *F*(1,58) = 6.08, *p* = .017, due to faster responses in female than male participants. However, an interaction between these factors was not found, *F*(1,58) = 0.31, *p* = .582.

IES: ANOVA showed a main effect of Congruency, *F*(1,58) = 11.71, *p* < .0001, due to lower IES scores on congruent than incongruent trials, and of Participant Sex, *F*(1,58) = 6.70, *p* = .012, due to lower scores for female than male participants. However, an interaction between these factors was not found, *F*(1,58) = 0.49, *p* = .488.

**Experiment 3**

Mean and standard deviation for accuracy (Acc), response times (RTs) and inverse efficiency scores (IES) for the congruent (C) and incongruent (I) condition in male and female participants of Experiment 3.

|  | | | | | | | | | | | | | | | |
| --- | --- | --- | --- | --- | --- | --- | --- | --- | --- | --- | --- | --- | --- | --- | --- |
|  |  |  |  |  |  |  |  |  |  |  |  |  |  |  |  |
|  | | **Sex** | | **Acc C** | | **Acc I** | | **RTs C** | | **RTs I** | | **IES C** | | **IES I** | |
| Mean |  | Female |  | 94.625 |  | 94.625 |  | 595.87 |  | 597.53 |  | 635.89 |  | 640.22 |  |
|  |  | Male |  | 95.917 |  | 97.292 |  | 632.60 |  | 633.33 |  | 659.14 |  | 650.63 |  |
| Standard deviation |  | Female |  | 7.4795 |  | 8.6050 |  | 92.999 |  | 90.204 |  | 127.99 |  | 138.56 |  |
|  |  | Male |  | 3.0957 |  | 2.4363 |  | 89.032 |  | 86.456 |  | 86.946 |  | 84.687 |  |
|  | | | | | | | | | | | | | | | |

Accuracy: ANOVA did not show a main effect of Congruency, *F*(1,58) = 3.86, *p* = .054, or of Participant Sex, *F*(1,58) = 1.70, *p* = .197, and no interaction between these factors, *F*(1,58) = 3.86, *p* = .054.

RTs: ANOVA did not show a main effect of Congruency, *F*(1,58) = 0.10, *p* = .753, or of Participant Sex, *F*(1,58) = 2.52, *p* = .118, and no interaction between these factors, *F*(1,58) = 0.02, *p* = .903.

IES: ANOVA did not show a main effect of Congruency, *F*(1,58) = 0.17, *p* = .681, or of Participant Sex, *F*(1,58) = 0.35, *p* = .557, and no interaction between these factors, *F*(1,58) = 1.62, *p* = .209.

**Experiment 4**

Mean and standard deviation for accuracy (Acc), response times (RTs) and inverse efficiency scores (IES) for the congruent (C) and incongruent (I) condition in male and female participants of Experiment 4.

|  | | | | | | | | | | | | | | | |
| --- | --- | --- | --- | --- | --- | --- | --- | --- | --- | --- | --- | --- | --- | --- | --- |
|  |  |  |  |  |  |  |  |  |  |  |  |  |  |  |  |
|  | | **Sex** | | **Acc C** | | **Acc I** | | **RTs C** | | **RTs I** | | **IES C** | | **IES I** | |
| Mean |  | Female |  | 94.833 |  | 95.667 |  | 606.34 |  | 595.71 |  | 640.02 |  | 622.81 |  |
|  |  | Male |  | 94.750 |  | 94.750 |  | 554.74 |  | 555.35 |  | 586.10 |  | 586.50 |  |
| Standard deviation |  | Female |  | 5.5197 |  | 4.3516 |  | 95.919 |  | 86.587 |  | 94.905 |  | 84.258 |  |
|  |  | Male |  | 3.3541 |  | 3.6020 |  | 69.739 |  | 80.416 |  | 75.990 |  | 84.264 |  |
|  | | | | | | | | | | | | | | | |

Accuracy: ANOVA did not show a main effect of Congruency, *F*(1,58) = 0.91, *p* = .345, or of Participant Sex, *F*(1,58) = 0.24, *p* = .625, and no interaction between these factors, *F*(1,58) = 0.91, *p* = .345.

RTs: ANOVA did not show a main effect of Congruency, *F*(1,58) = 1.93, *p* = .170, but a main effect of Participant Sex, *F*(1,58) = 4.66, *p* = .035, due to faster responses from male than female participants. No interaction between these factors was found, *F*(1,58) = 2.44, *p* = .124.

IES: ANOVA did not show a main effect of Congruency, *F*(1,58) = 3.66, *p* = .061, but a main effect of Participant Sex, *F*(1,58) = 4.39, *p* = .041, due to lower inverse efficiency scores in male than female participants. The interaction between these factors also approached significance, *F*(1,58) = 4.02, *p* = .050. Tukey HSD test showed that inverse efficiency scores were lower on incongruent trials than congruent trials in female participants, *p* = .037. No other differences reached significance, all *ps* => .08.
